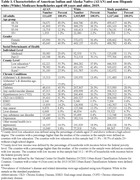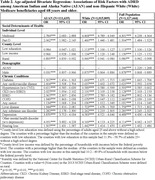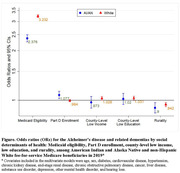# Associations between Social Determinants of Health and Alzheimer’s Disease and Related Dementia among American Indian and Alaska Native and White Medicare Beneficiaries

**DOI:** 10.1002/alz.092107

**Published:** 2025-01-09

**Authors:** Manxi Yang, Ruqoyat Abdulsalam, Richard Adjei‐Boateng, Lingling Li, Spero Manson, Joan O'Connell, Luohua Jiang

**Affiliations:** ^1^ University of California Irvine, Irvine, CA USA; ^2^ University of Colorado Denver, Denver, CO USA; ^3^ University of Colorado Anschutz Medical Campus, Aurora, CO USA

## Abstract

**Background:**

Alzheimer’s disease and related dementias (ADRD) present a growing challenge in the US and has emerged as a significant health concern in American Indian and Alaska Native (AI/AN) communities. ADRD prevalence and risk factors among AI/AN adults remain poorly understood and plagued by inconsistent findings. This study addressed this knowledge gap by examining the associations of social determinants of health (SDOH) with ADRD between AI/AN and non‐Hispanic White (White) populations.

**Method:**

AI/AN and a 5% random sample of White adults aged 68 and above enrolled in fee‐for‐service (FFS) Medicare in 2019 were included in this study. We used the 2019 Medicare Beneficiary Summary File to obtain data on beneficiaries’ demographics, chronic conditions, and health coverage. We obtained county‐level data from the 2019 Area Health Resource File and the National Center for Health Statistics. Medicaid eligibility and Part D enrollment were used as proxies for individual‐level SDOH. County‐level measures were defined by the percentage of households with incomes below the federal poverty level, the percentage of adults without a high school degree, and county rurality. Separately for AI/AN and White populations, we calculated crude and age‐adjusted prevalence of ADRD and used logistic regressions to examine associations between ADRD and SDOH, controlling for demographics and chronic conditions.

**Result:**

Among 111,635 AI/AN and 1,015,809 White Medicare beneficiaries included, the ADRD prevalence was 15.6% (age‐adjusted) and 13.4% respectively (OR = 1.24, 95% CI:1.22, 1.27) (Table 2). After multivariate adjustment, the odds ratio of AI/AN for ADRD was not statistically significant (OR = 1.006, 95% CI: 0.980, 1.033). As shown in the Figure, Medicaid eligibility was associated with a substantially higher odds of ADRD among both AI/AN (OR = 2.38, 95% CI: 2.26, 2.50) and White individuals (OR = 3.23, 95% CI: 3.17, 3.29). Part D enrollment was associated with higher odds of ADRD among AI/AN individuals only while county‐level income and education were associated with higher odds of ADRD among White individuals only. Additionally, rurality was associated with lower odds of ADRD in both populations.

**Conclusion:**

SDOH are potentially modifiable factors, therefore, a deeper understanding of their effects on ADRD can inform future interventions aimed at reducing ADRD prevalence and disparities.